# Visual field changes after vitrectomy with internal limiting membrane peeling for epiretinal membrane or macular hole in glaucomatous eyes

**DOI:** 10.1371/journal.pone.0177526

**Published:** 2017-05-18

**Authors:** Shunsuke Tsuchiya, Tomomi Higashide, Kazuhisa Sugiyama

**Affiliations:** Department of Ophthalmology, Kanazawa University Graduate School of Medical Science, Kanazawa, Japan; Massachusetts Eye & Ear Infirmary, Harvard Medical School, UNITED STATES

## Abstract

**Purpose:**

To investigate visual field changes after vitrectomy for macular diseases in glaucomatous eyes.

**Methods:**

A retrospective review of 54 eyes from 54 patients with glaucoma, who underwent vitrectomy for epiretinal membrane (ERM; 42 eyes) or macular hole (MH; 12 eyes). Standard automated perimetry (Humphrey visual field 24–2 program) was performed and analyzed preoperatively and twice postoperatively (1^st^ and 2nd sessions; 4.7 ± 2.5, 10.3 ± 3.7 months after surgery, respectively). Postoperative visual field sensitivity at each test point was compared with the preoperative value. Longitudinal changes in mean visual field sensitivity (MVFS) of the 12 test points within 10° eccentricity (center) and the remaining test points (periphery), best-corrected visual acuity (BCVA), intraocular pressure (IOP), and ganglion cell complex (GCC) thickness, and the association of factors with changes in central or peripheral MVFS over time were analyzed using linear mixed-effects models. In addition, 45 eyes from 45 patients without glaucoma who underwent vitrectomy for epiretinal membrane (ERM; 34 eyes) or macular hole (MH; 11 eyes) were similarly examined and statistically analyzed (control group).

**Results:**

In glaucomatous eyes, visual field test points changed significantly and reproducibly; two points deteriorated only at the center and twelve points improved only at the periphery. Central MVFS decreased (p = 0.03), whereas peripheral MVFS increased postoperatively (p = 0.010). In the control group, no visual field test points showed deterioration, and central MVFS did not change significantly after vitrectomy. BCVA improved, GCC thickness decreased, and IOP did not change postoperatively in both groups. The linear mixed-effects models identified older age, systemic hypertension, longer axial length, and preoperative medication scores of ≥2 as risk factors for central MVFS deterioration in glaucomatous eyes.

**Conclusions:**

Visual field sensitivity within 10° eccentricity may deteriorate after vitrectomy for ERM or MH in glaucomatous eyes.

## Introduction

Recent advances in pars plana vitrectomy (PPV) have brought about more successful anatomical and functional outcomes in the surgical treatment of epiretinal membrane (ERM) and macular hole (MH). Microincision vitrectomy surgery for ERM showed less inflammation, faster recovery, and better visual outcomes [1]. Peeling of the internal limiting membrane (ILM) may improve outcomes related to visual acuity and anatomic parameters, and reduce recurrence rates in ERM and MH surgeries [2].

However, a number of studies have identified safety concerns pertaining to retinal damage given the manipulation of the retinal surface of the macula required for membrane removal. In particular, the peeling of the ILM [3–5] and the use of indocyanine green for visualization of the ILM [6] may have adverse effects on the central visual fields (VFs).

Macular disorders sometimes coexist with glaucoma, especially in elderly patients. Asrani et al. reported that more than 10% of glaucomatous eyes had an ERM responsible for the artifacts in macular scans by optical coherence tomography (OCT) [7]. Aging has been identified as a representative risk factor for both ERM and glaucoma [8,9]. Given that the prevalence of both diseases in a Japanese population was reported to be approximately 5% [8,10], the number of glaucomatous eyes that undergo PPV for macular diseases may be underestimated and is expected to increase in aging societies. In patients with glaucoma, the macula is the site of surgical manipulations during PPV for ERM or MH, and is often affected even in early stages of the disease. About 50% of the hemifields examined by a 10–2 VF program for the macula already showed abnormalities in patients with early-stage glaucoma [11].

Therefore, PPV for macular diseases can be a serious threat to the central VF in glaucomatous eyes. However, there has been only one case series with 7 patients (7 glaucomatous eyes) that reported a significant decrease in mean deviation (MD) on the Humphrey VF test after PPV for macular diseases [12]. The purpose of the present study was to examine if PPV has negative effects on the VF in glaucomatous eyes and to identify factors associated with VF changes.

## Subjects and methods

### Study design

This was a retrospective observational study with consecutive cases of open-angle glaucoma that underwent PPV for MH or ERM at Kanazawa University Hospital from May 2010 to October 2015. In addition, non-glaucomatous patients with MH or ERM who underwent PPV at our institution were prospectively enrolled in this study (control group). The study protocol was approved by the Institutional Review Board of Kanazawa University Hospital and adhered to the tenets of the Declaration of Helsinki. Written informed consent was obtained from all participants.

### Preoperative examinations

The patients underwent routine preoperative ophthalmic evaluations including measurement of best-corrected visual acuity (BCVA) with a 5-meter Landolt chart, slit-lamp examination, intraocular pressure (IOP) measurements using a Goldmann applanation tonometer, axial length measurement (OA-1000, TOMEY, Tokyo, Japan), gonioscopy, dilated fundus examination, fundus photography, standard automated perimetry (SAP, Humphrey visual field Analyzer II, 24–2 Swedish interactive threshold algorithm, Humphrey-Zeiss instrument, Dublin, CA), and spectral-domain OCT examination using a RS-3000 Retina Scan (Nidek Inc., Gamagori, Aichi, Japan).

Glaucoma was diagnosed by abnormalities in the optic disc (enlarged cupping, neuroretinal rim thinning and retinal nerve fiber layer defects) and reproducible VF defects corresponding to the optic disc changes. A glaucomatous VF defect was defined as follows: 1) a cluster of three points with a probability <5% on a pattern deviation map in at least one hemifield and including at least one point with a probability <1%, or a cluster of two points with a probability <1%; 2) glaucoma hemifield test results outside normal limits; and 3) pattern standard deviation <5%. The eyes in the control group had no glaucomatous abnormalities in the optic disc.

### Inclusion and exclusion criteria

The following inclusion criteria were used: normal anterior segment, normal open-angle by gonioscopy, reliable SAP results (fixation losses <33%, false positive rate <20% and false negative rate <20%), and good quality OCT images (no imaging artifacts, signal strength index >6). Patients with other vision threatening diseases such as corneal diseases, visually significant cataract, uveitis, diabetic retinopathy, and macular degenerative diseases were excluded. Eyes with an indeterminate appearance of the optic disc were excluded from the study.

### Surgical procedures

Surgeries were performed by a single surgeon (T.H.) using a 23-, 25- or 27-gauge pars plana vitrectomy with the Accurus 800CS (Alcon Surgical, Fort Worth, TX) or Constellation Vision System (Alcon Surgical). Triamcinolone acetonide (TA; MaQaid^®^, Wakamoto Pharmaceutical, Tokyo, Japan) was used to visualize the vitreous [13] in all cases, and for facilitating posterior vitreous detachment (PVD) induction in eyes not having a PVD. In eyes with ERM, the ERM was directly grasped and peeled with end-gripping forceps. TA was also utilized for peeling the ILM in both MH [14] and ERM [15]. TA was re-injected over the macula after ERM removal, and excess TA was removed by aspiration with a backflush needle to identify the remnants of the ILM. In more recent cases, brilliant blue G (BBG; ILM-Blue^®^, DORC International, Zuidland, The Netherlands) was used for ILM staining [16]. The ILM was directly grasped and peeled with end-gripping forceps within the area of approximately 10° eccentricity from the fovea. ERM and ILM peeling were usually started in the superior quadrant. Cataract surgery was combined with vitrectomy for phakic eyes. Fluid-gas exchange (FGX) and intraocular gas tamponade with 20% SF_6_ were performed for all eyes with MH or eyes with ERM in which intraoperative retinal breaks were found during a thorough peripheral vitreous shaving.

### Analysis of SAP and other test results

**Fig 1:**
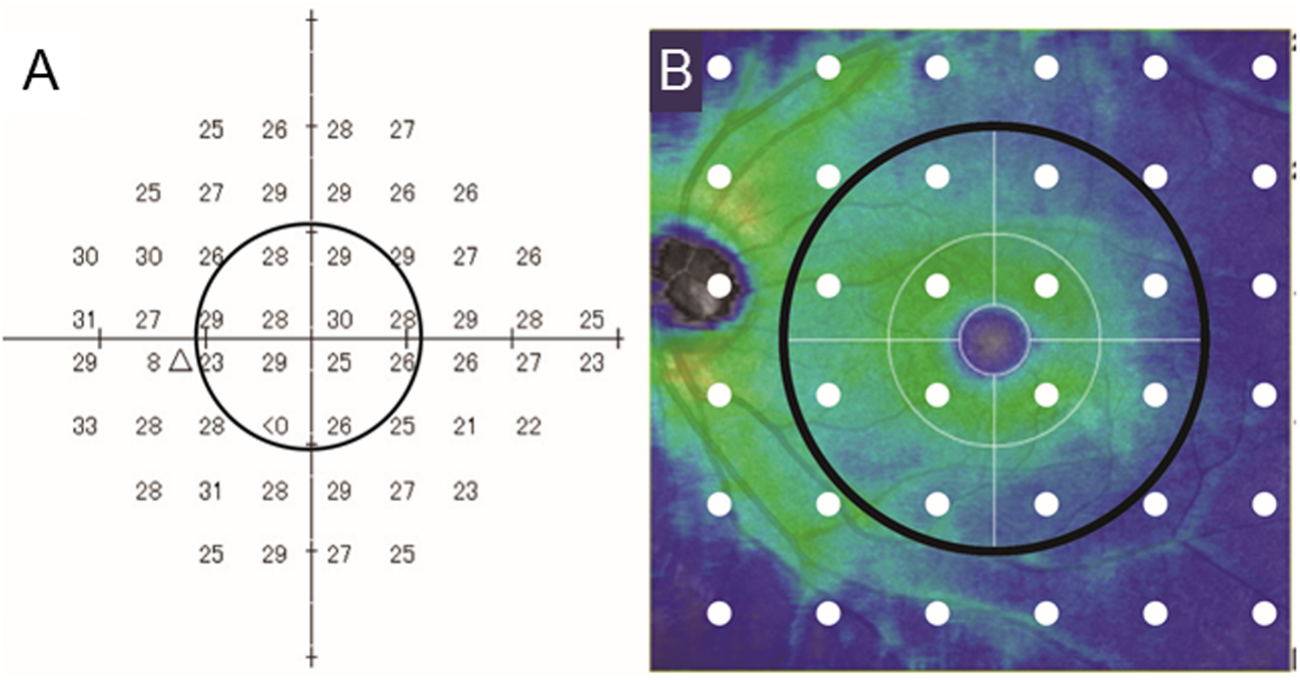
Humphrey visual field (HFA) 24–2 test points and corresponding retinal locations. (A) An example of HFA 24–2 test results. The circle indicates the area within 10° eccentricity. In this case, central and peripheral mean visual field sensitivities were 27.8 dB and 27.4 dB, respectively. (B) A retinal thickness map generated by optical coherence tomography showing the relationship between visual field test points and the area of retinal thickness measurement. White dots show HFA 24–2 test points. The black circle (diameter = 6 mm) corresponds to the area of 10° eccentricity.

SAP was performed postoperatively and the results from three consecutive sessions (one preoperative and two postoperative sessions) were analyzed. In addition to MD and pattern standard deviation (PSD), central and peripheral mean VF sensitivity (MVFS, expressed in dB) were calculated. The areas of the central and peripheral VF were within and outside of 10° eccentricity, respectively. The 12 test points were within the central VF area (Fig 1A). The anti-logged values of the visual sensitivity at 52 non–blind spot locations were averaged separately for the central or peripheral areas and were then logged to convert back to a dB scale to calculate MVFS [17]. A sensitivity value of <0 dB was treated as -2 dB in accordance with the calculation for total deviation.

Visual acuity and OCT measurements were performed during the visits for VF testing. For the postoperative IOP, data were obtained during the postoperative SAP sessions. The average thickness of the ganglion cell complex (GCC; retinal nerve fiber layer [RNFL] + ganglion cell layer + inner plexiform layer) of a 6 mm circular area in the macula, which corresponds to the area within 10° eccentricity, was obtained from the OCT software (Fig 1B).

### Evaluation of dissociated optic nerve fiber layer (DONFL) appearance

A DONFL appearance, which was originally reported by Tadayoni et al. [18], consists of numerous arcuate striae slightly darker than the surrounding retinal surface. Determination of the presence or absence of a DONFL was based on postoperative blue-filter fundus photographs taken after three months or more.

### Statistical analysis

BCVA was converted into the logarithm of the minimal angle of resolution (logMAR) format. The demographic data were compared between eyes with ERM and MH or between eyes with and without glaucoma using Fisher’s exact tests, Chi-square tests, Student's t-tests, or Mann-Whitney U tests as indicated. The VF sensitivity at each of the 52 non–blind spot locations at two postoperative sessions were compared independently with preoperative values using a Wilcoxon signed rank test. Left eye data were converted to a right eye format. The locations where p values were <5% after the Bonferroni correction in both postoperative sessions were regarded as having a significant change.

The postoperative changes in BCVA, IOP, GCC thickness and VF parameters in each group were analyzed using linear mixed-effects models in which the fixed effects included SAP session order, disease type (ERM or MH), and interaction between them, and random effects were patient-specific intercepts and slopes (i.e. trend over time after vitrectomy). The influence of glaucoma on the time course of each parameter was examined for all eyes using linear mixed-effects models in which the fixed effects included SAP session order, groups (glaucoma or control), and interaction between them, and random effects were patient-specific intercepts and slopes. The mixed-effects models with patient-specific random intercepts and slopes were also employed to identify factors which had significant influence on central and peripheral MVFS over time in eyes with glaucoma. The following variables were examined: time [i.e. the interval (months) between vitrectomy and SAP], gender, age, history of systemic hypertension (HT), axial length, disease type, number of preoperative glaucoma medications (medication scores), preoperative BCVA, preoperative IOP, preoperative GCC thickness, simultaneous cataract surgery, BBG usage, FGX, and DONFL appearance. Medication scores were divided into 3 categories: 0, 1, and 2 or more. The final multivariate model was created using backwards selection by successively removing variables with least-significant effects or interactions with time until only those variables with significant effects or interactions with time remained [19,20]. The final model also contained time as an independent variable.

All statistical analyses were performed using SPSS version 23.0 (SPSS Inc., Chicago, IL) and Stata software (version 14.1; StataCorp, TX). The statistical significance level was set as P <0.05.

## Results

The study included 54 eyes from 54 patients with glaucoma. Patient characteristics are shown in Table 1. The age of the patients at PPV was 68.1 ± 8.0 (mean ± standard deviation) years old. Fourteen patients (25.9%) had HT. Fifty-one eyes (94.4%) had primary open angle glaucoma and three eyes had exfoliation glaucoma. Preoperative BCVA (logMAR) was 0.19 ± 0.20 and was significantly better in eyes with ERM (p = 0.01). Preoperative MD and PSD were -7.8 ± 5.6 dB and 6.8 ± 4.1 dB, respectively. Preoperative IOP and medication scores were 13.7 ± 3.0 mmHg and 1.1 ± 1.2, respectively. Preoperative GCC thickness was 114.5 ± 21.4 μm. Forty-two eyes (77.8%) underwent combined cataract surgery. FGX was performed in 17 eyes (31.5%) and was significantly more frequent in eyes with MH (p <0.001). BBG was used in 22 eyes (40.7%). DONFL appearance was detected in 9 eyes (16.7%). The first and second postoperative SAP sessions were 4.7 ± 2.5 and 10.3 ± 3.7 months after surgery, respectively.

**Table 1:** Comparison of factors between glaucomatous eyes with ERM and MH.

Factors	Total (n = 54)	ERM (n = 42)	MH (n = 12)	P value
Male/female	19/35	17/25	2/10	0.12*
Age (years)	68.1 ± 8.0	68.2 ± 8.3	67.8 ± 7.1	0.88^†^
Right/left eye	22/32	16/26	6/6	0.34*
Hypertension	14	10	4	0.37*
Visual acuity (logMAR)	0.19 ± 0.20	0.16 ± 0.16	0.32 ± 0.27	0.01^†^
Axial length (mm)	25.0 ± 2.1	24.8 ± 2.1	25.5 ± 2.3	0.35^‡^
Intraocular pressure (mmHg)	13.7 ± 3.0	13.6 ± 2.9	13.9 ± 3.3	0.76^†^
Medication score	1.1 ± 1.2	1.2 ± 1.2	0.8 ± 0.9	0.30^‡^
Mean deviation (dB)	-7.8 ± 5.6	-8.4 ± 5.7	-5.8 ± 5.0	0.11^‡^
Pattern standard deviation (dB)	6.8 ± 4.1	7.1 ± 4.1	6.0 ± 4.2	0.32^‡^
GCC thickness (μm)	114.5 ± 21.4	116.3 ± 22.3	108.4 ± 17.5	0.32^‡^
Combined cataract surgery	42	33	9	0.54*
FGX	17	5	12	<0.001*
BBG usage	22	20	2	0.05*
DONFL appearance	9	5	4	0.10*

ERM = epiretinal membrane; MH = macular hole; logMAR = logarithm of the minimal angle of resolution; GCC = ganglion cell complex; FGX = fluid-gas exchange; BBG = brilliant blue G; DONFL = dissociated optic nerve fiber layer.

*Fisher’s exact test

^†^Two sample t-test

^‡^Mann-Whitney U test

In addition, 45 eyes from 45 patients without glaucoma who underwent vitrectomy for epiretinal membrane (ERM; 34 eyes) or macular hole (MH; 11 eyes) were enrolled as a control group. Demographic data comparing the glaucoma group with the control group are shown in the S1 Table. Age, disease types (ERM/ MH), MH stages, and the time points of the first and second postoperative SAP sessions were not significantly different between the two groups. Comparisons of demographics between non-glaucomatous eyes with ERM and MH are shown in the S2 Table. Preoperative BCVA (logMAR) was significantly better in eyes with ERM (p <0.001). Preoperative MD was significantly better in eyes with MH (p = 0.02).

**Fig 2:**
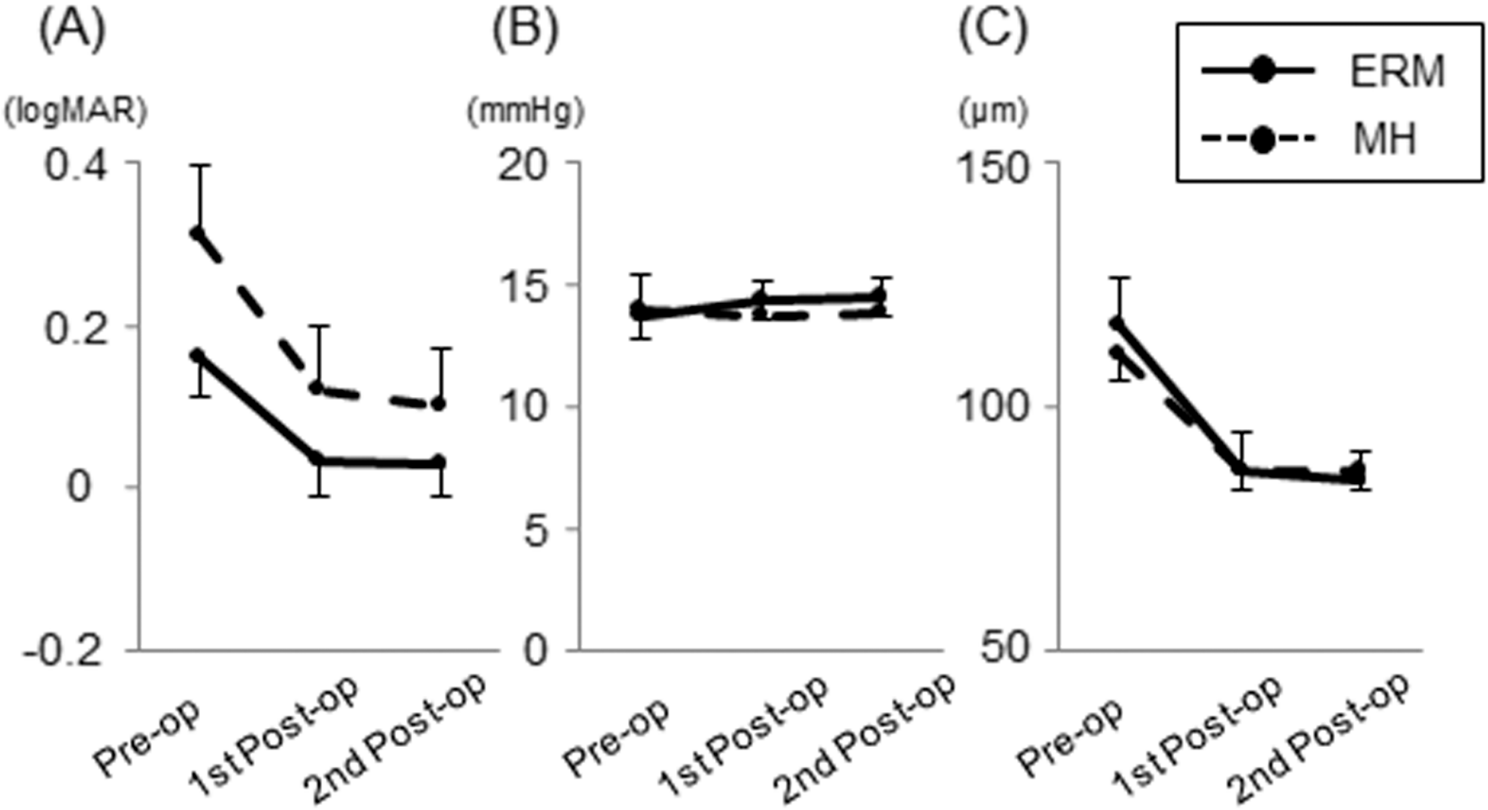
Longitudinal changes in parameters of glaucomatous eyes with epiretinal membrane (ERM) and macular hole (MH). (A) Best-corrected visual acuity (logMAR). (B) Intraocular pressure. (C) Ganglion cell complex thickness. Estimated marginal means from the linear mixed-effects models are plotted for each session of visual field testing. Error bars = 95% confidence interval.

**Fig 3:**
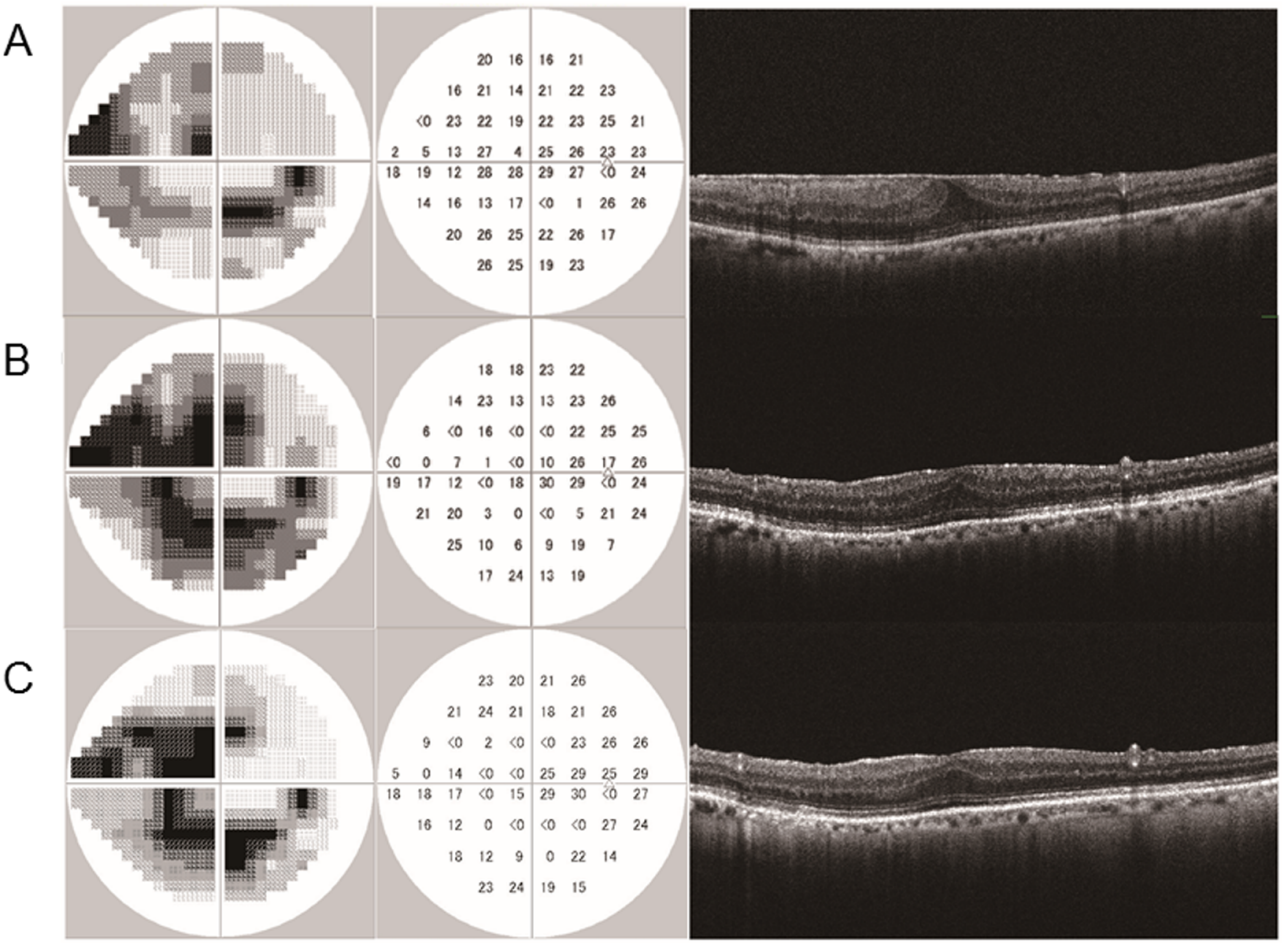
A representative case of epiretinal membrane (ERM) in a glaucomatous eye which showed worsening of the central visual field after vitrectomy. A 77-year-old man underwent vitrectomy in his right eye. (A) The preoperative visual field test showed glaucomatous changes (i.e. superior and inferior arcuate defects and superior paracentral scotoma). The mean deviation (MD) and pattern standard deviation (PSD) were -10.2 dB and 8.7 dB, respectively. The optical coherence tomography (OCT) image shows an ERM. (B) A visual field test at the first postoperative session (7 months after surgery) revealed decreased sensitivity especially in the central area. The MD and PSD were -16.7 dB and 11.7 dB, respectively. The ERM was successfully removed and his visual acuity had improved to 0.8 from 0.6. (C) A visual field test and an OCT image at the second postoperative session (10 months after surgery). The MD and PSD were -16.2 dB and 12.9 dB, respectively. The worsening of his central visual field sensitivity persisted. (Left) gray-scale plots. (Middle) visual field sensitivity (dB) at each test point. (Right) OCT images from a vertical scan through the fovea.

In glaucomatous eyes, the mean BCVA (logMAR) significantly improved after surgery (p <0.001, Fig 2A). IOP was well controlled with or without topical glaucoma medications during the study periods in all cases, and IOP did not show significant changes after surgery (P = 0.88; Fig 2B). GCC thickness significantly decreased after surgery (P <0.001; Fig 2C). A representative case is presented in Fig 3. The postoperative course of visual acuity, IOP and GCC thickness in the control group was similar to the glaucoma group (S1 Fig). There were no significant intraoperative nor postoperative complications in all cases.

### Postoperative VF changes in glaucomatous eyes

**Fig 4:**
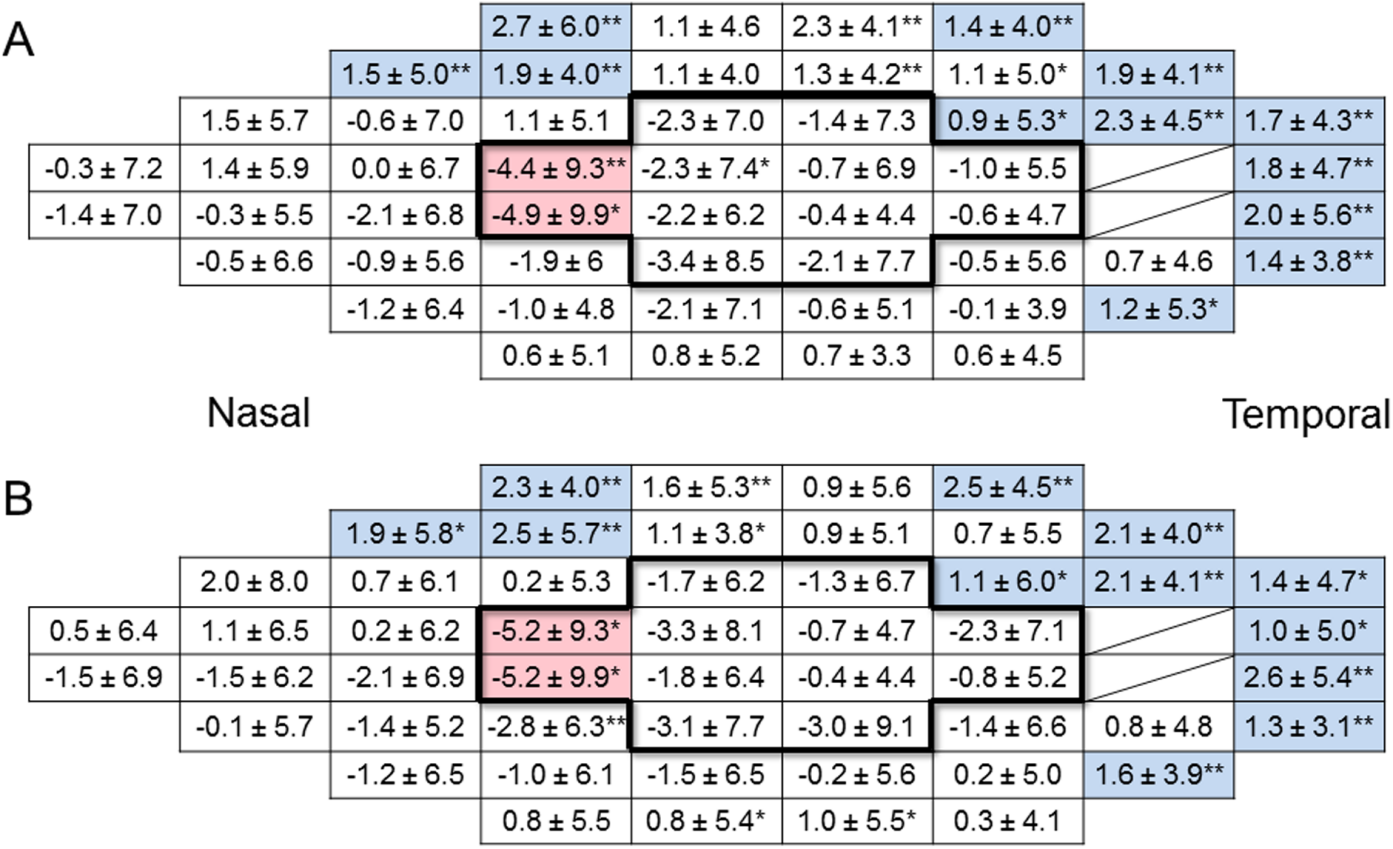
Mean retinal sensitivity changes at each test point of the Humphrey visual field 24–2 program in glaucomatous eyes. The 12 central boxes surrounded by bold lines are located within 10° eccentricity. The red boxes indicate significantly deteriorated points and the blue boxes indicate points that were significantly ameliorated at both postoperative test sessions. (A) 1st postoperative session. (B) 2nd postoperative session. Data are shown as the mean ± standard deviation (dB). *P <0.05, **P <0.01. The diagonal lines indicate blind spots.

**Fig 5:**
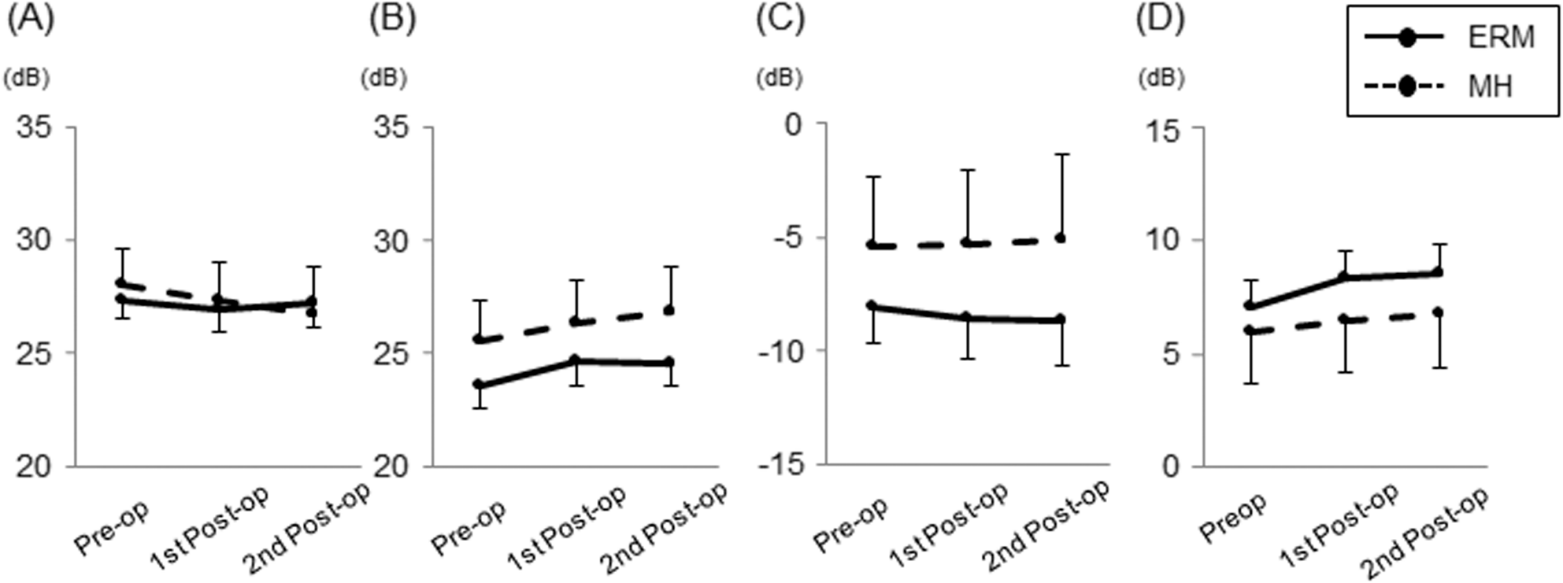
Longitudinal changes in visual field parameters of glaucomatous eyes with epiretinal membrane (ERM) and macular hole (MH). **(**A) Central mean visual field sensitivity. (B) Peripheral mean visual field sensitivity. (C) Mean deviation. (D) Pattern standard deviation. Estimated marginal means from the linear mixed-effects models are plotted for each session of visual field testing. Error bars = 95% confidence interval.

Two test points which were located in the nasal area between 5° and 10° eccentricity showed significant deterioration in both postoperative SAP sessions (Fig 4). In contrast, there were 12 significantly improved test points in both SAP sessions. All improved points were located outside of 10° eccentricity. The VF was then divided into central and peripheral areas by 10° eccentricity. The postoperative central MVFS decreased significantly after surgery (P = 0.03; Fig 5A), while postoperative peripheral MVFS improved significantly after surgery (P = 0.01; Fig 5B). Postoperative MD and PSD were not significantly different from baseline (P = 0.71, 0.17, respectively; Fig 5C and 5D). Disease type had no significant effect on longitudinal changes in these parameters.

### Postoperative VF changes in the control group

No test points showed significant deterioration at both postoperative SAP sessions, while 15 test points were significantly ameliorated after surgery (S2 Fig). All improved points were located outside of 10° eccentricity. The central MVFS remained stable (P = 0.09; S3A Fig), while the peripheral MVFS significantly improved after surgery (P = 0.02; S3B Fig). Postoperative MD increased significantly (P = 0.02; S3C Fig), whereas PSD was unchanged (P = 0.07; S3D Fig). Disease type had no significant effect on longitudinal changes in these parameters except for PSD which showed a more negative trend (i.e. improvement) in eyes with MH than in eyes with ERM (P = 0.014 for the interaction between disease type and SAP session order).

### Comparisons of longitudinal changes in parameters between glaucoma and control groups

**Fig 6:**
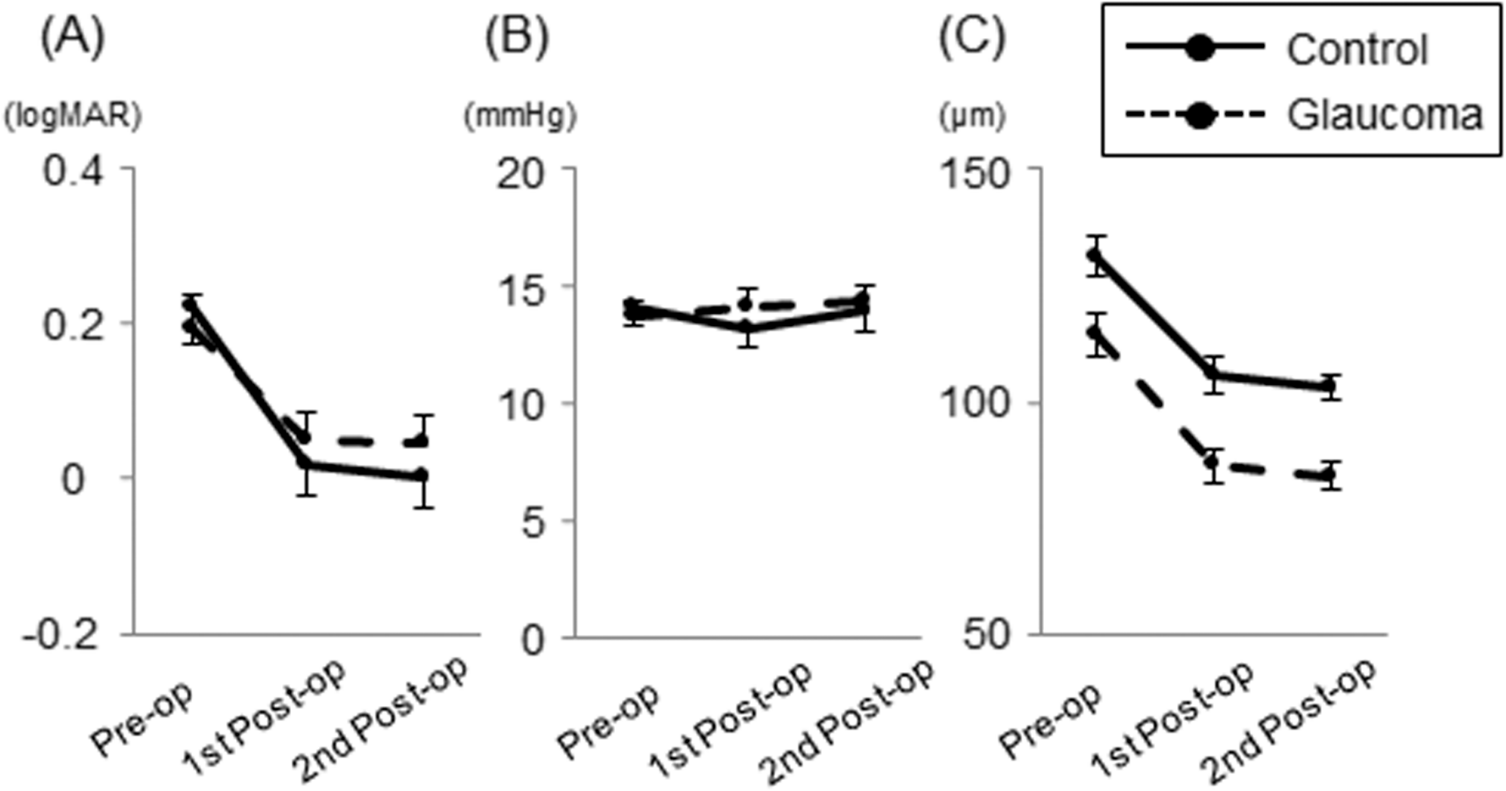
Comparisons of longitudinal changes in parameters between glaucoma and control groups. (A) Best-corrected visual acuity (logMAR). (B) Intraocular pressure. (C) Ganglion cell complex thickness. Estimated marginal means from the linear mixed-effects models are plotted for each session of visual field testing. Error bars = 95% confidence interval.

**Fig 7:**
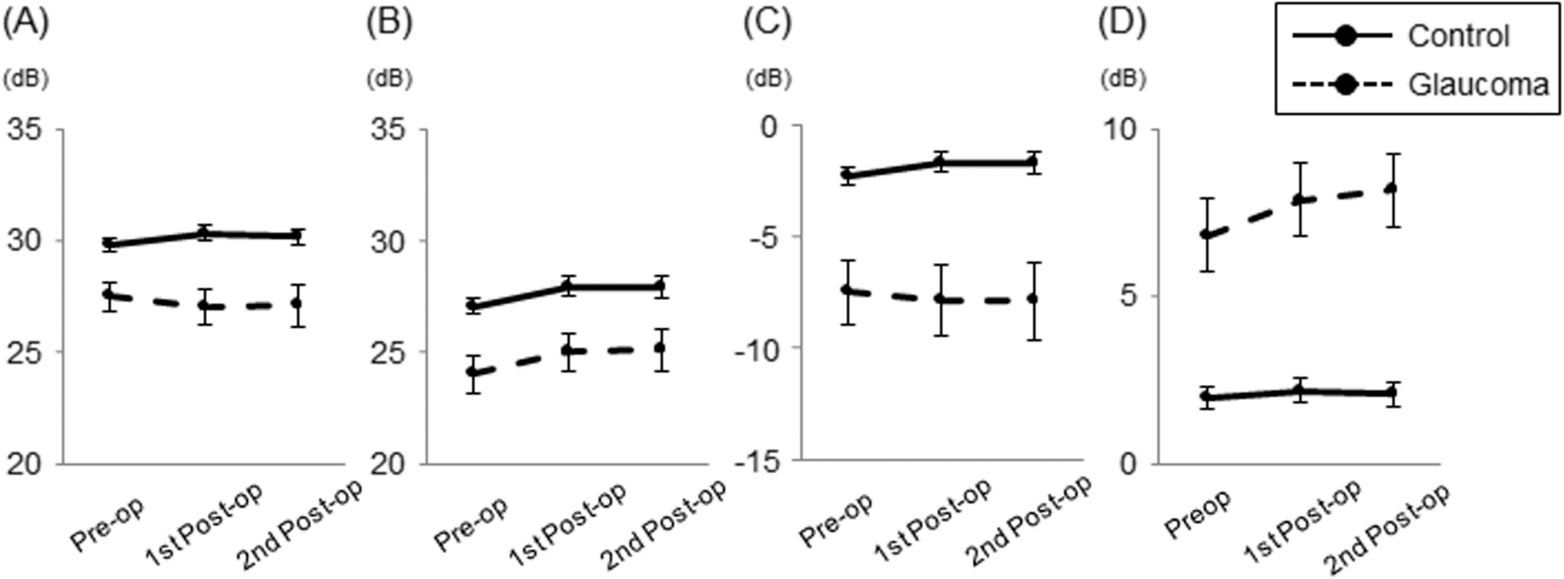
Comparisons of longitudinal changes in visual field parameters between glaucoma and control groups. (A) Central mean visual field sensitivity; (B) Peripheral mean visual field sensitivity; (C) Mean deviation; (D) Pattern standard deviation. Estimated marginal means from the linear mixed-effects models are plotted for each session of visual field testing. Error bars = 95% confidence interval.

Table 2 shows the results of linear mixed-effects model analysis for all eyes to examine the influence of glaucoma on the time course of each parameter, and Figs 6 and 7 illustrate the differences in longitudinal changes of parameters between glaucoma and control groups. All VF parameters were significantly worse in the glaucoma group than in the control group (P <0.001). GCC thickness was significantly thinner in glaucomatous eyes than in control eyes (p <0.001). BCVA and IOP were not significantly different between groups. The interaction between glaucoma and time (i.e. SAP session order) in Table 2 indicates whether glaucoma had significant influence on the time course of each parameter. Regarding the VF parameters, glaucoma had a significantly negative interaction with time in the central MVFS and MD (coefficient, -0.37, -0.56; 95% confidence interval [CI], -0.71 to -0.03, -0.95 to -0.16; p = 0.035, 0.005, respectively), but not in the peripheral MVFS. Namely, glaucoma was significantly associated with a negative trend (i.e. worsening) of the central MVFS and MD. Furthermore, glaucoma had a significantly positive interaction with time in PSD (coefficient, 0.63; 95% CI, 0.33 to 0.93; p <0.001). In addition, glaucoma had a marginally significant association (p = 0.045) with a positive trend (i.e. worsening) of BCVA (logMAR).

**Table 2:** Influence of glaucoma on longitudinal changes in parameters of all eyes (n = 99).

	Variables		
	Glaucoma	Time*	Glaucoma x time*
Parameters	Coef. (SE), P value	Coef. (SE), P value	Coef. (SE), P value
BCVA (logMAR)	-0.02 (0.04), 0.62	-0.11 (0.01), <0.001	0.04 (0.02), 0.045
Intraocular pressure (mmHg)	-0.15 (0.54), 0.78	-0.13 (0.19), 0.49	0.45 (0.25), 0.07
GCC thickness (μm)	-16.8 (3.40), <0.001	-14.3 (1.36), <0.001	-1.14 (1.88), 0.54
Central MVFS (dB)	-2.58 (0.42), <0.001	0.18 (0.13), 0.16	-0.37 (0.17), 0.035
Peripheral MVFS (dB)	-3.03 (0.51), <0.001	0.45 (0.11), <0.001	0.07 (0.15), 0.64
Mean deviation (dB)	-5.36 (0.84), <0.001	0.31 (0.14), 0.03	-0.56 (0.20), 0.005
Pattern standard deviation (dB)	4.94 (0.62), <0.001	0.05 (0.11), 0.63	0.63 (0.15), <0.001

Coef. = coefficients; SE = standard errors; BCVA = best corrected visual acuity; logMAR = logarithm of the minimal angle of resolution; GCC = ganglion cell complex; MVFS = mean visual field sensitivity.

*Time indicates the SAP session order. The interactions with time (x time) indicate the effect of glaucoma on the slope of changes over time, and a negative value corresponds to a declining trend over time.

### Significant factors influencing central and peripheral MVFS over time in glaucomatous eyes

Linear mixed model analysis incorporating the between-patient random effects was performed to explore the factors associated with changes in central and peripheral MVFS over time in eyes with glaucoma (Table 3). Older age and longer axial length had a significantly negative interaction with time in the central MVFS (coefficient, -0.01, -0.04; 95% confidence interval [CI], -0.02 to -0.002, -0.06 to -0.007; p = 0.01, 0.02, respectively). Namely, these factors were significantly associated with a negative postoperative trend (i.e. worsening) of central MVFS. In addition, the coexistence of systemic hypertension was significantly associated with worsening of both central and peripheral MVFS over time (coefficient, -0.11, 0.095; 95% CI, -0.2 to -0.005, -0.19 to -0.006; p = 0.04, 0.04, respectively). Patients treated with ≥2 medications preoperatively not only had significantly lower baseline central and peripheral MVFS, but were also significantly more likely to show deterioration in central and peripheral MVFS compared to those untreated (coefficient, -0.17, 0.10; 95% CI, -0.28 to -0.05, -0.20 to -0.004; p = 0.005, 0.04, respectively). Eyes with DONFL appearance had a significantly higher baseline central MVFS than those without (coefficient, 2.13; 95% CI, 0.33 to 3.94; p = 0.02), and were significantly associated with a negative trend for peripheral MVFS over time (coefficient, -0.13; 95% CI, -0.25 to -0.003; p = 0.04). A larger logMAR value (i.e. worse BCVA) was associated with a positive trend (i.e. improvement) in peripheral MVFS (coefficient, 0.21; 95% CI, 0.03 to 0.40; p = 0.03). Disease type, simultaneous cataract surgery, BBG usage and FGX were insignificant for changes in either central or peripheral MVFS over time.

**Table 3:** Factors influencing central and peripheral MVFS over time in glaucomatous eyes.

Factors	Central MVFS	Peripheral MVFS
	Coef. (SE), P value	Coef. (SE), P value
Intercept (dB)	27.9 (0.6), <0.001	27.2 (1.0), <0.001
Time (months)*	0.06 (0.04), 0.18	0.14 (0.06), 0.02
Age^†^ (years)	-0.09 (0.05), 0.095	-0.14 (0.05), 0.005
Age^†^ x time	-0.01 (0.004), 0.01	-0.004 (0.003), 0.16
Hypertension	0.38 (0.70), 0.58	-1.09 (0.84), 0.19
Hypertension x time	-0.11 (0.05), 0.04	-0.10 (0.046), 0.04
ERM vs. MH	NA	-2.1 (0.94), 0.03
ERM vs. MH x time	NA	0.018 (0.06), 0.74
Preop. Med. 1 vs. 0	-1.13 (0.75), 0.13	-0.57 (0.88), 0.52
Preop. Med. 2 vs. 0	-2.05 (0.74), 0.006	-2.76 (0.88), 0.002
Preop. Med. 1 x time	-0.02 (0.06), 0.72	-0.02 (0.05), 0.73
Preop. Med. 2 x time	-0.17 (0.06), 0.005	-0.10 (0.05), 0.04
Axial length^†^ (mm)	0.07 (0.19), 0.71	NA
Axial length^†^ x time	-0.04 (-0.01), 0.02	NA
Preop. BCVA (logMAR)^†^	NA	-2.2 (1.98), 0.27
Preop. BCVA^†^ x time	NA	0.21 (0.10), 0.03
DONFL	2.13 (0.92), 0.02	0.10 (1.05), 0.92
DONFL x time	-0.12 (0.07), 0.09	-0.12 (0.06), 0.04

MVFS = mean visual field sensitivity; ERM = epiretinal membrane; MH = macular hole; Coef. = coefficients; SE = standard errors; NA = not applicable due to elimination from the model; Preop. Med. = number of glaucoma medications divided into 3 categories: 0, 1, and 2 or more; BCVA = best corrected visual acuity; logMAR = logarithm of the minimal angle of resolution; DONFL = dissociated optic nerve fiber layer.

*Time indicates the interval (months) between vitrectomy and visual field testing. The interactions with time (x time) indicate the effect of the variable on the slope of MVFS changes over time. Negative values correspond to MVFS deterioration over time.

^†^Variables were centered at their mean values.

## Discussion

Two SAP test points in the nasal area between 5° and 10° eccentricity showed significant and sustained deterioration after PPV with ILM peeling for ERM or MH in glaucomatous eyes, whereas none of the test points showed significant worsening in eyes without glaucoma. Comparative studies showed that in non-glaucomatous eyes, central retinal sensitivity was significantly lower in eyes with ILM peeling after PPV for MH or ERM than those without [4,5]. The nasal SAP test points corresponded to the temporal quadrant of the macula where paracentral scotomas were most likely to occur and retinal sensitivity was relatively lower after PPV for MH with ILM peeling in non-glaucomatous eyes [3,21]. The temporal quadrant of the macula has the thinnest RNFL in normal subjects [22], and therefore may be most vulnerable to mechanical stress by ILM peeling. Our results indicate that the vulnerability of this macular area is higher in eyes with glaucoma than those without. From a clinical perspective, the decrease in retinal sensitivity may be more serious in glaucomatous eyes, especially at advanced stages, than in non-glaucomatous eyes.

Although MD and PSD did not show significant changes after surgery, central MVFS deteriorated over time in contrast to the improvement in peripheral MVFS in eyes with glaucoma. In contrast, central MVFS remained unchanged postoperatively in the control group. The worsening of the central MVFS was directly associated with glaucoma given that glaucoma had significantly negative influence on the longitudinal changes in the central MVFS. Among the various factors examined, older age and longer axial length were significantly associated with a worsening of retinal sensitivity only within 10° eccentricity where membrane peeling was performed. The susceptibility of retinal ganglion cells to damage has been shown to increase with age in rodent glaucoma-related models [23,24]. Age-related ganglion cell loss in human eyes has also been demonstrated histologically [25] and was corroborated by OCT studies showing age-related thinning of circumpapillary RNFL and inner retinal layers in the macula [26]. In regards to axial length, the inner retinal thickness (i.e. ganglion cell layer plus inner plexiform layer) within 10° eccentricity in the macula is thinner in eyes with longer axial length [27]. Taken together, these results suggest that mechanical damage by membrane peeling may be more pronounced in eyes with a more vulnerable, thinner central macula.

Medication scores significantly influenced both central and peripheral MVFS at baseline and over the time course. Patients whose eyes have lower target IOP due to advanced glaucoma or faster VF progression tend to have multiple medications. The negative MVFS trend in eyes with higher medication scores indicates that VF loss after PPV may be more likely to occur in advanced glaucoma or include glaucomatous VF progression.

Systemic hypertension was identified as a significant risk for worsening of both central and peripheral MVFS. Use of systemic antihypertensives was also an independent risk factor associated with VF progression in the Low-pressure Glaucoma Treatment Study [28]. Patients with systemic hypertension may suffer from compromised ocular blood flow due to arteriosclerosis. Noma et al. reported that blood flow velocity in the perifoveal capillaries was lower in hypertensive patients [29]. Recent studies showed that optic nerve head blood flow significantly decreased during PPV in response to epinephrine in the infusion solution and an elevated infusion pressure [30,31]. Therefore, hypertensive patients may have more compromised blood flow in the optic nerve head during PPV, resulting in exacerbation of VF damage.

DONFL appearance was not associated with worsening of central MVFS. Although earlier reports detected no significant loss of retinal sensitivity in the DONFL area after PPV for MH [32–34], Nukada et al. reported significantly lower central retinal sensitivity within the DONFL regions than outside of them [35]. Spectral-domain OCT showed that inner retinal defects corresponding to the arcuate striae may extend beyond the RNFL into the ganglion cell layer and inner plexiform layer. ICG staining, which was reported to be toxic to retinal ganglion cells [36] and to cause thinning of the RNFL [37], was used in most cases in these previous studies, however it was not used in the present study. In a spectral-domain OCT study of eyes which underwent PPV for MH and ERM using BBG staining, the DONFL area was characterized by dimpling caused by trauma to the Müller cells along with the regenerative growth of the Müller cell processes rather than actual dissociation of nerve fiber bundles [38]. Thus, DONFL appearance after PPV in glaucomatous eyes may not be a sign of worsening of central VF due to RNFL damage. In contrast, central MVFS at baseline was significantly higher in eyes with DONFL appearance. Given that the RNFL in glaucomatous eyes is already thin, the dimpled appearance of the DONFL may become more difficult to detect in eyes with more advanced glaucoma.

In the present study, 42 and 41 phakic patients (78 and 91%) underwent PPV combined with cataract surgery in the glaucoma and control groups, respectively. Previous reports showed that cataract surgery affects VF parameters in glaucomatous eyes, and that MD improved significantly after cataract extraction [39,40]. In our study, MD in eyes without glaucoma was ameliorated postoperatively although none of the patients had a visually significant cataract. Similarly, a prospective study had demonstrated that MD from the 24–2 program showed improvement in eyes with ERM 1 year after PPV [41]. Since PPV was performed without cataract surgery in the study, the improvement is not attributable to cataract extraction. In contrast, MD in glaucomatous eyes remained stable after surgery. Worsening of central MVFS may result in unchanged MD by offsetting the improvement in peripheral MVFS. The effect of cataract extraction on PSD remains controversial. While a few reports did not show any PSD changes [42,43], other reports showed PSD deterioration after cataract surgery in patients with glaucoma [39,40]. A diffuse sensitivity loss due to cataract may mask focal defects in glaucomatous VFs. Although the linear mixed-effects models did not show significant PSD changes after surgery, the postoperative improvement in peripheral MVFS indicates possible positive effects by PPV and/or cataract extraction on this part of the VF.

There are several limitations of our study. First, the study design for the glaucoma patients was retrospective and the interval between PPV and VF sessions was not the same in all cases. We enrolled consecutive cases to reduce selection bias, and employed mixed-effects models to account for patient-specific variation in the timing of VF testing. Furthermore, the time points of postoperative VF testing in the control group were determined to match those in the glaucoma group.

The sample size of the study was small especially for eyes with MH or eyes that underwent PPV only. Although the disease type and simultaneous cataract surgery did not appear to be significant determinants of postoperative VF changes, further studies with a larger sample size are needed to address these issues. The optimal strategy for VF testing to study changes after PPV is debatable. The 6° grid of the 24–2 program could miss and/or underestimate the sensitivity loss in the macula after PPV compared to the 2° grid of the 10–2 program examining the VF area within 10° eccentricity. Microperimetry may have better reproducibility especially in eyes with unstable fixation owing to the automated real-time fundus tracking and alignment. However, the microperimetry test area is usually within 10° eccentricity [3,6,21,32–35]. The wider test area of the 24–2 program enabled us to compare VF changes within and outside of 10° eccentricity (i.e. within and outside of the area of membrane peeling).

In conclusion, we investigated VF changes after PPV for ERM or MH in glaucomatous eyes in comparison with non-glaucomatous eyes. There were two test points within 10° eccentricity where visual field sensitivity significantly and reproducibly decreased after PPV only in glaucomatous eyes. The worsening of the central VF was directly associated with glaucoma. Furthermore, aging, longer axial length, higher medication scores and systemic hypertension may be risk factors for the deterioration of central VF sensitivity. The risk of central VF deterioration should be considered prior to PPV in patients with macular diseases and coexisting glaucoma.

## Supporting information

S1 Fig Longitudinal changes in parameters in non-glaucomatous eyes with epiretinal membrane (ERM) and macular hole (MH). (A) Best-corrected visual acuity (logMAR). (B) Intraocular pressure. (C) Ganglion cell complex thickness. Estimated marginal means from the linear mixed-effects models are plotted for each session of visual field testing. Error bars = 95% confidence interval.
(TIF)

S2 Fig Mean retinal sensitivity changes at each test point of the Humphrey visual field 24–2 program (non-glaucomatous eyes). The 12 central boxes surrounded by bold lines are located within 10° eccentricity. The blue boxes indicate points that were significantly ameliorated at both postoperative test sessions. (A) 1st postoperative session. (B) 2nd postoperative session. Data are shown as the mean ± standard deviation (dB). *P<0.05, **P<0.01. The diagonal lines indicate blind spots.
(TIF)

S3 Fig Longitudinal changes in visual field parameters of non-glaucomatous eyes with epiretinal membrane (ERM) and macular hole (MH). (A) Central mean visual field sensitivity. (B) Peripheral mean visual field sensitivity. (C) Mean deviation. (D) Pattern standard deviation. Estimated marginal means from the linear mixed-effects models are plotted for each session of visual field testing. Error bars = 95% confidence interval.
(TIF)

S1 Table Comparison of factors between eyes with and without glaucoma. (DOCX)

S2 Table Comparison of factors between non-glaucomatous eyes with ERM and MH. (DOCX)
