# Supplementary figures and images for: Visual field changes after vitrectomy with internal limiting membrane peeling for epiretinal membrane or macular hole in glaucomatous eyes
Source: PLoS One. 2017 May 18;12(5):e0177526. doi: 10.1371/journal.pone.0177526 (PMC5436669; doi:10.1371/journal.pone.0177526)

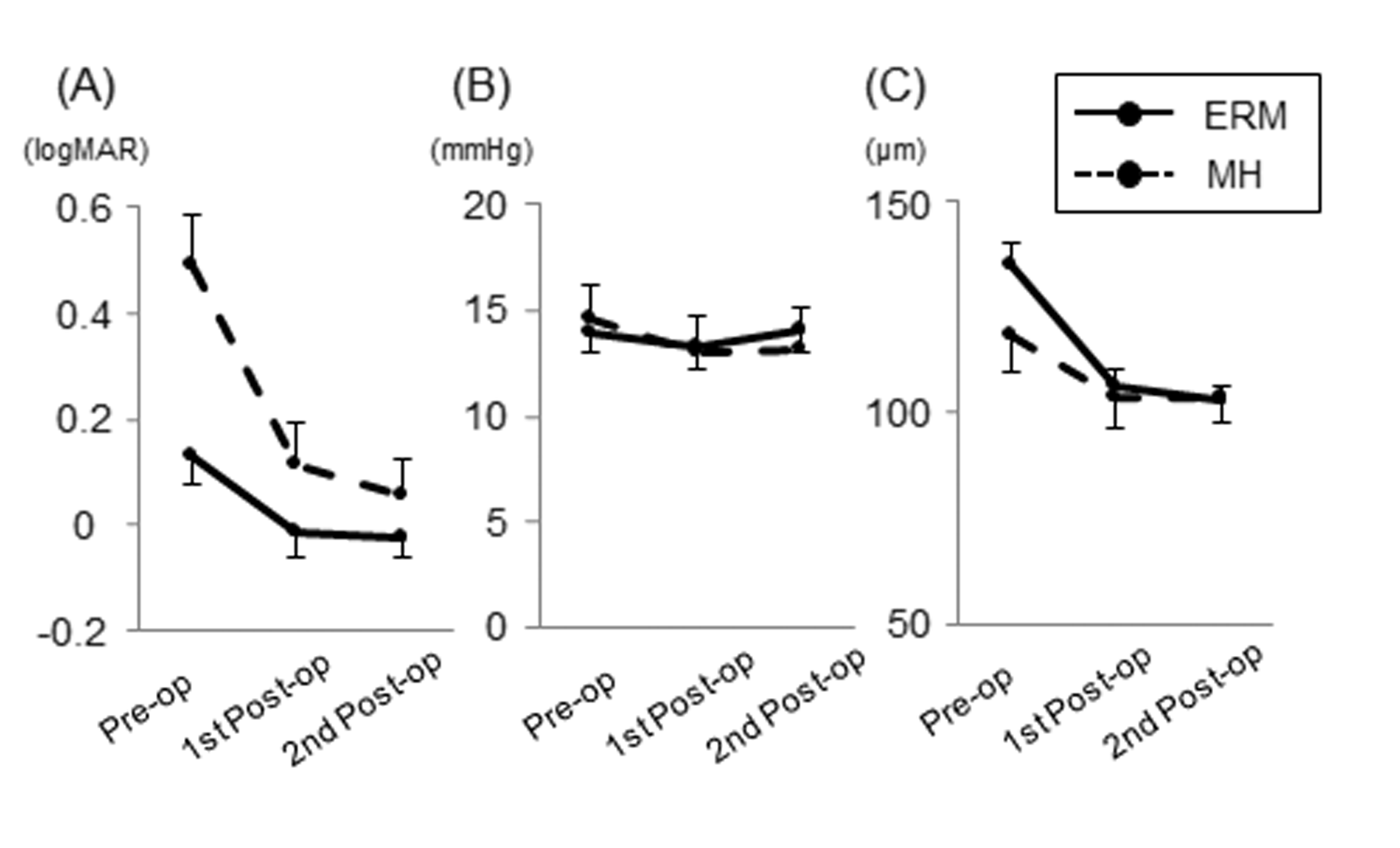

Supplement: S1 Fig — (A) Best-corrected visual acuity (logMAR). (B) Intraocular pressure. (C) Ganglion cell complex thickness. Estimated marginal means from the linear mixed-effects models are plotted for each session of visual field testing. Error bars = 95% confidence interval. (TIF) [file pone.0177526.s001.tif]

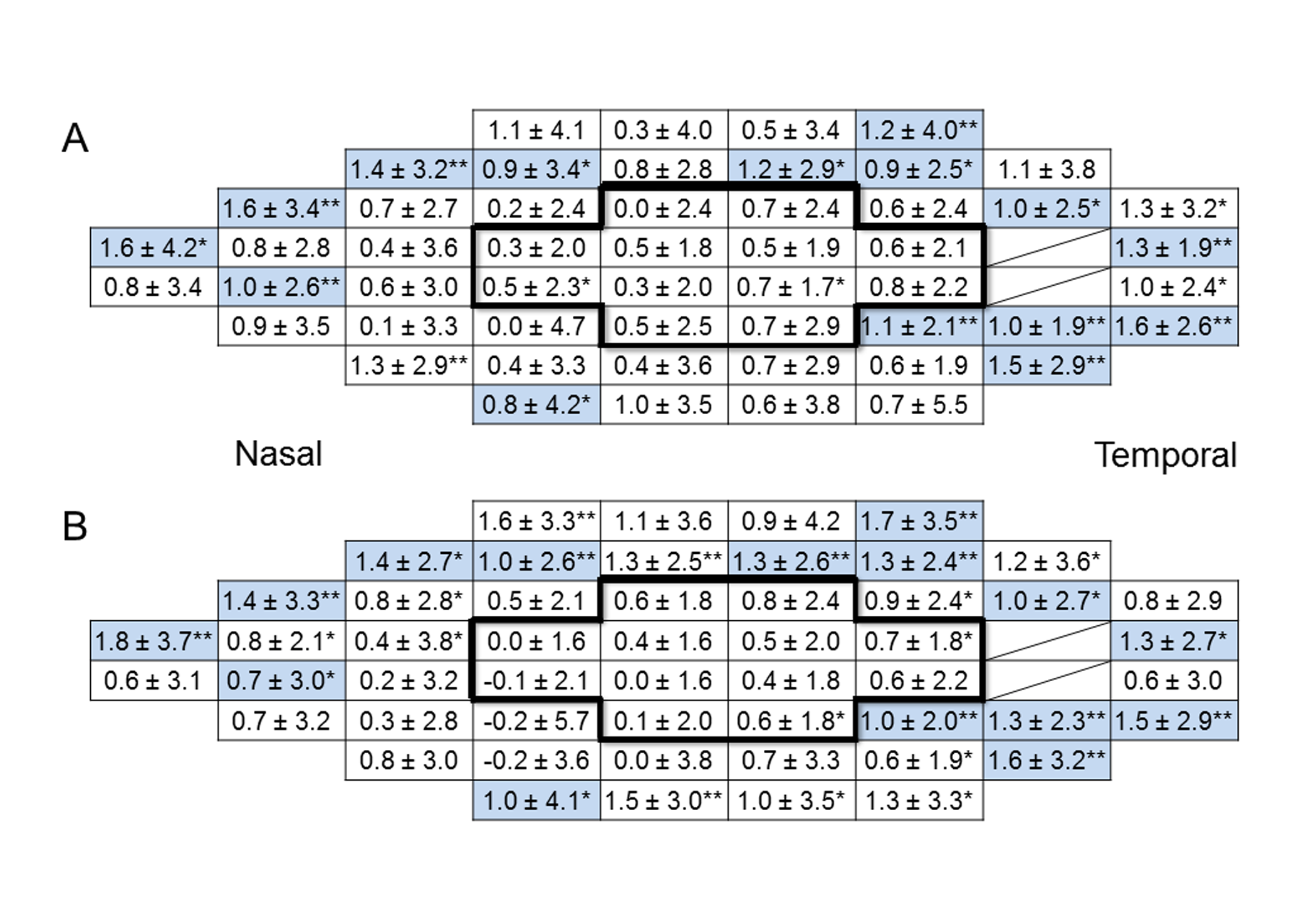

Supplement: S2 Fig — The 12 central boxes surrounded by bold lines are located within 10° eccentricity. The blue boxes indicate points that were significantly ameliorated at both postoperative test sessions. (A) 1st postoperative session. (B) 2nd postoperative session. Data are shown as the mean ± standard deviation (dB). *P<0.05, **P<0.01. The diagonal lines indicate blind spots. (TIF) [file pone.0177526.s002.tif]

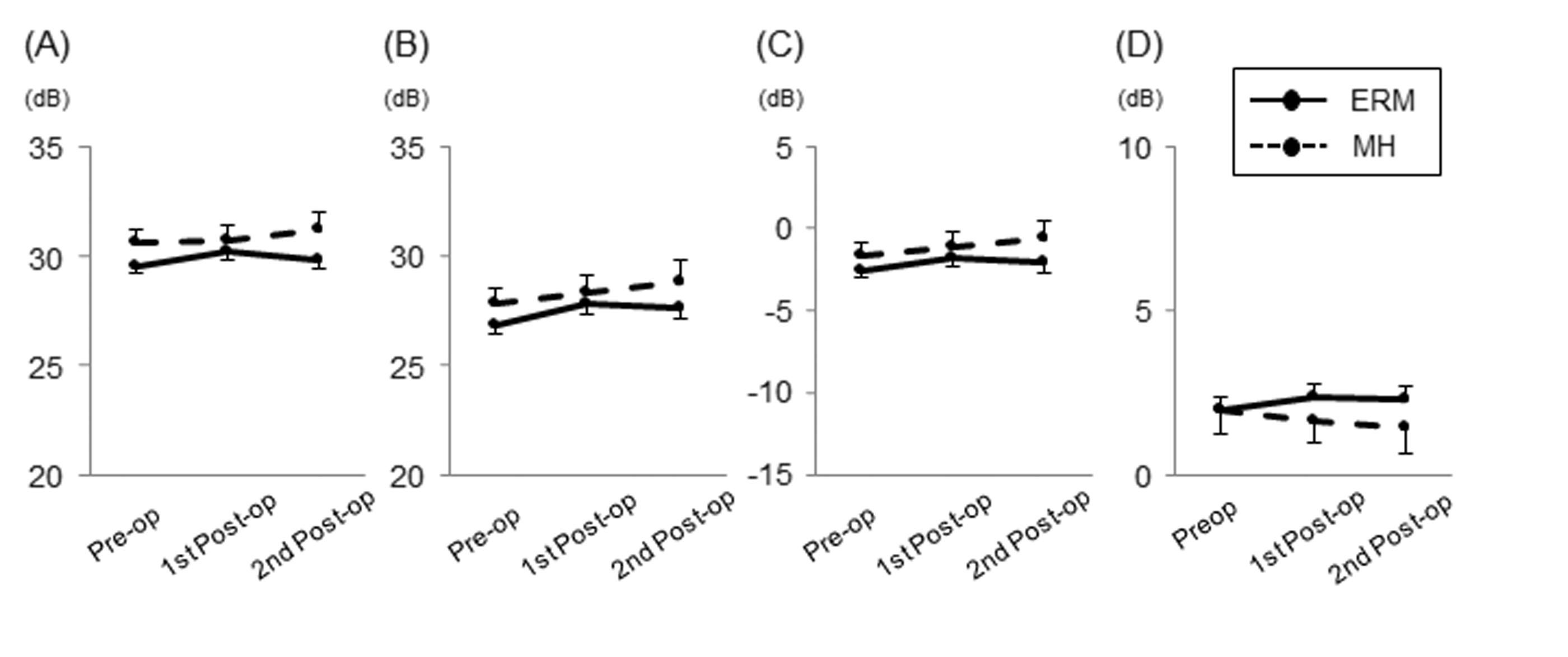

Supplement: S3 Fig — (A) Central mean visual field sensitivity. (B) Peripheral mean visual field sensitivity. (C) Mean deviation. (D) Pattern standard deviation. Estimated marginal means from the linear mixed-effects models are plotted for each session of visual field testing. Error bars = 95% confidence interval. (TIF) [file pone.0177526.s003.tif]
