# Supplementary material for: Visual field changes after vitrectomy with internal limiting membrane peeling for epiretinal membrane or macular hole in glaucomatous eyes
Source: PLoS One. 2017 May 18;12(5):e0177526. doi: 10.1371/journal.pone.0177526 (PMC5436669; doi:10.1371/journal.pone.0177526)
Supplement: S1 Table — (DOCX) [file pone.0177526.s004.docx]

**S1 Table. Comparison of factors between eyes with and without glaucoma**

| Factors | Glaucoma (n = 54) | Control (n = 45) | P value |
| --- | --- | --- | --- |
| Male/ female | 19/ 35 | 14/ 31 | 0.67* |
| Age (years) | 68.1 ± 8.0 | 65.6 ± 6.0 | 0.09^†^ |
| ERM/ MH | 42/ 12 | 34/ 11 | 0.79* |
| MH stages (stage 2/ 3/ 4) | 1/ 3/ 8 | 1/ 7/ 3 | 0.15* |
| Right/ left eye | 22/ 32 | 26/ 19 | 0.09* |
| Hypertension | 14 | 11 | 0.87* |
| Visual acuity (logMAR) | 0.19 ± 0.20 | 0.22 ± 0.25 | 0.97^†^ |
| Axial length (mm) | 25.0 ± 2.1 | 23.9 ± 1.3 | 0.02^‡^ |
| Intraocular pressure (mmHg) | 13.7 ± 3.0 | 14.2 ± 2.8 | 0.42^†^ |
| Med. score | 1.1 ± 1.2 | 0 | 0.0001^‡^ |
| Mean deviation (dB) | -7.8 ± 5.6 | -2.7 ± 1.6 | <0.001^‡^ |
| Pattern standard deviation (dB) | 6.8 ± 4.1 | 2.1 ± 1.1 | <0.001^‡^ |
| GCC thickness (µm) | 114.5 ± 21.4 | 131.2 ± 18.9 | <0.001^‡^ |
| Combined cataract surgery | 42 | 41 | 0.07* |
| FGX | 17 | 12 | 0.60* |
| BBG usage | 22 | 44 | <0.001* |
| DONFL appearance | 9 | 10 | 0.49* |
| The first postoperative SAP session (months after surgery) | 4.7 ± 2.5 | 3.5 ± 0.9 | 0.23^‡^ |
| The second postoperative SAP session (months after surgery) | 10.3 ± 3.7 | 8.8 ± 3.4 | 0.27^‡^ |

ERM = epiretinal membrane; MH = macular hole; logMAR = logarithm of the minimal angle of resolution; GCC = ganglion cell complex; FGX = fluid-gas exchange; BBG = brilliant blue G; DONFL = dissociated optic nerve fiber layer; SAP: standard automated perimetry. *Chi-Square test, ^†^Two sample t-test, ^‡^Mann-Whitney U test.
