# Supplementary material for: Visual field changes after vitrectomy with internal limiting membrane peeling for epiretinal membrane or macular hole in glaucomatous eyes
Source: PLoS One. 2017 May 18;12(5):e0177526. doi: 10.1371/journal.pone.0177526 (PMC5436669; doi:10.1371/journal.pone.0177526)
Supplement: S2 Table — (DOCX) [file pone.0177526.s005.docx]

**S2 Table. Comparison of factors between non-glaucomatous eyes with ERM and MH**

| Factors | Total (n = 45) | ERM (n=34) | MH (n=11) | P value |
| --- | --- | --- | --- | --- |
| Male/ female | 14/ 31 | 9/ 25 | 5/ 6 | 0.28* |
| Age (years) | 65.6 ± 6.0 | 65.7 ± 6.2 | 65.1 ± 5.5 | 0.76^†^ |
| Right/ left eye | 26/ 19 | 17/ 17 | 9/ 2 | 0.09* |
| Hypertension | 11 | 9 | 2 | 0.71* |
| Visual acuity (logMAR) | 0.22 ± 0.25 | 0.13 ± 0.19 | 0.48 ± 0.23 | <0.001^‡^ |
| Axial length (mm) | 23.9 ± 1.3 | 24.0 ± 1.3 | 23.5 ± 1.2 | 0.16^‡^ |
| Intraocular pressure (mmHg) | 14.2 ± 2.8 | 14.0 ± 2.9 | 14.6 ± 2.7 | 0.52^†^ |
| Mean deviation (dB) | -2.7 ± 1.6 | -3.0 ± 1.6 | -1.7 ± 0.9 | 0.02^†^ |
| Pattern standard deviation (dB) | 2.1 ± 1.1 | 2.1 ± 1.2 | 2.2 ± 1.0 | 0.55^‡^ |
| GCC thickness (µm) | 131.2 ± 18.9 | 135.3 ± 19.6 | 118.4 ± 8.8 | <0.001^†^ |
| Combined cataract surgery | 41 | 30 | 11 | 0.56* |
| FGX | 12 | 1 | 11 | <0.001* |
| BBG usage | 44 | 34 | 10 | 0.24* |
| DONFL appearance | 10 | 6 | 4 | 0.23* |

ERM = epiretinal membrane; MH = macular hole; logMAR = logarithm of the minimal angle of resolution; GCC = ganglion cell complex; FGX = fluid-gas exchange; BBG = brilliant blue G; DONFL = dissociated optic nerve fiber layer.

*Fisher’s exact test, ^†^Two sample t-test, ^‡^Mann-Whitney U test.
